# Identification of a Predominantly Interferon-λ-Induced Transcriptional Profile in Murine Intestinal Epithelial Cells

**DOI:** 10.3389/fimmu.2017.01302

**Published:** 2017-10-16

**Authors:** Tharini A. Selvakumar, Sudeep Bhushal, Ulrich Kalinke, Dagmar Wirth, Hansjörg Hauser, Mario Köster, Mathias W. Hornef

**Affiliations:** ^1^Hannover Medical School, Institute for Medical Microbiology and Hospital Epidemiology, Hannover, Germany; ^2^Research Group Model Systems for Infection and Immunity, Helmholtz Centre for Infection Research (HZI), Braunschweig, Germany; ^3^Institute for Experimental Infection Research, TWINCORE, Centre for Experimental and Clinical Infection Research, A Joint Venture between the Helmholtz Centre for Infection Research and the Hannover Medical School, Hannover, Germany; ^4^Department of Experimental Hematology, Hannover Medical School, Hannover, Germany; ^5^Institute for Medical Microbiology, RWTH Aachen University Hospital, Aachen, Germany

**Keywords:** interferon-lambda, intestinal epithelium, interleukin 28 receptor, transcription, gastrointestinal tract

## Abstract

Type I (α and β) and type III (λ) interferons (IFNs) induce the expression of a large set of antiviral effector molecules *via* their respective surface membrane receptors. Whereas most cell types respond to type I IFN, type III IFN preferentially acts on epithelial cells and protects mucosal organs such as the lung and gastrointestinal tract. Despite the engagement of different receptor molecules, the type I and type III IFN-induced signaling cascade and upregulated gene profile is thought to be largely identical. Here, we comparatively analyzed the response of gut epithelial cells to IFN-β and IFN-λ_2_ and identified a set of genes predominantly induced by IFN-λ_2_. We confirm the influence of epithelial cell polarization for enhanced type III receptor expression and demonstrate the induction of predominantly IFN-λ_2_-induced genes in the gut epithelium *in vivo*. Our results suggest that IFN-λ_2_ targets the epithelium and induces genes to adjust the antiviral host response to the requirements at mucosal body sites.

## Introduction

The interferon (IFN) family of cytokines acts to confer protection against various pathogens. They are categorized into three different types. Whereas the type II IFN, IFN-γ, plays a key role in the host response to intracellular bacteria and parasites, members of the type I IFNs-α and β and the more recently discovered type III IFNs-λ mediate antiviral protection (1–3). Type I and type III IFNs are secreted by a wide range of different cell types upon innate immune stimulation. Differences exist with respect to their transcriptional regulation due to a distinct transcription factor requirement explaining discrepancies in their expression kinetics (4–7). Type I and III IFNs share low amino acid similarity (15–20%) and bind to structurally very different heterodimeric receptor complexes comprised of the IFN-α receptor (IFNAR) 1 and 2 chain as well as the IFN-λ receptor (IFN-λR) 1 and the IL-10 receptor (IL-10R)β chain, respectively (2, 3). The type I IFN receptor is ubiquitously expressed by all nucleated cells although differences in the expression level and functional sensitivity have been reported (8, 9). By contrast, the type III IFN receptor is restricted to epithelial cells at mucosal body sites and distinct immune cell subpopulations such as for example polymorphonuclear cells (PMNs) (10–16). Consistently, epithelial cells of the gastrointestinal, respiratory, and reproductive tract were identified as primary targets for type III IFNs *in vivo* (8–11, 17–21). The type III IFN mediated effect on the epithelium of respiratory and gastrointestinal body surfaces thereby allow an early antiviral response in the absence of the systemic side effects and overt tissue inflammation (22).

Despite differences in their receptor utilization, both type I and type III IFNs engage the Jak/STAT signaling pathway leading to the formation of the IFN-stimulated gene factor (ISGF) 3 complex consisting of STAT1/2 heterodimers together with the interferon regulatory factor 9. ISGF3 translocates to the nucleus and binds to IFN-stimulated response elements in the promoter of so-called IFN-stimulated genes (ISGs) that ultimately generate the antiviral state. In addition to this canonical signaling, IFNAR and IFN-λR stimulation activates the mitogen-activated protein kinase pathways, i.e., the extracellular signal-regulated kinase (ERK)-1/2, the stress-activated protein kinase/c-Jun N-terminal kinase, and the p38 kinase as well as the phosphatidylinositol 3-kinase pathway *via* phosphorylation of Akt (12, 23). The functional contribution of these alternative signaling pathways *in vivo* has remained less well defined.

In accordance with the similarity of the induced signal transduction pathways, the spectrum of genes induced by the two types of IFNs is generally considered to be identical or very similar (12, 20, 24–30). This finding is consistent with the reported redundant or synergistic action of both types of IFN *in vivo* (17, 18, 20) and raises the question on the evolutionary benefit of the two distinct sets of antiviral IFNs and their respective receptors. One possible explanation is a quantitative difference in the cellular response and indeed *in vitro* studies suggested that the kinetics and magnitude of ISG induction differ between type I and type III IFN stimulation with type I IFN triggering a significantly faster and more potent transcriptional response (2, 3, 28, 29, 31, 32). However, IFN-λ was able to induce ISG expression and efficiently protect from viral infection of the intestinal and respiratory tract *in vivo* (8, 9, 17, 19, 21, 33). Another explanation might be previously undetected differences in the gene expression profile that shapes the IFN-λ response to better match the specific requirements of the mucosal antiviral host response. For example, IFN-λ may contribute to healing following mucosal tissue damage (34).

Comparative analyses of the transcriptional profile induced by type I versus type III IFN have so far been performed on hepatocytes, respiratory epithelial cells, lymphocytes, and total intestinal tissue and failed to identify IFN-λ-specific targets (12, 20, 24–30). The most discriminatory response between type I and type III IFN has, however, so far been reported at the intestinal epithelium which represents the entry port for many pathogenic viruses (9). We therefore took advantage of the recently established Mx2-luciferase transgenic gut epithelial IEC10 cells that exhibit many typical features of the intestinal epithelium and respond robustly to both type I and type III IFN (32). Comparative transcriptomic profiling of polarized intestinal epithelial cells identified a predominantly IFN-λ_2_-induced set of genes. Selected target genes were confirmed *in vivo* by an analysis of intestinal epithelial cells prepared from IFN-λ_2_ treated IFNAR^−/−^ mice, and the critical involvement of enterocyte polarization for IL-28R expression was demonstrated.

## Materials and Methods

### Ethics Statement

All animal experiments were performed in compliance with the German animal protection law (TierSchG) and approved by the local animal welfare committee Niedersächsisches Landesamt für Verbraucherschutz und Lebensmittelsicherheit Oldenburg, Germany. Mice were housed under specific pathogen-free conditions and handled in accordance with regulations defined by FELASA and the national animal welfare body GV-SOLAS.^1^

### Animals

B6.A2G-Mx1-IFNAR1^−/−^ mice lacking functional type I IFN receptors (IFNAR1^−/−^), B6.A2G-Mx1-IL28Rα^−/−^ mice carrying intact *Mx1* alleles, and lacking a functional type III IFN receptor (IL28Rα^−/−^) were bred at the Central Mouse Facility of the Helmholtz Centre for Infection Research, Braunschweig and described elsewhere (17).

### *In Vitro* Cell Culture

The intestinal epithelial cell line (IEC) Mx2Luc was generated from a transgenic mouse containing the firefly luciferase gene under control of the Mx2 promoter region as described earlier (32). IECs were cultured at 37°C, 5% CO_2_, 95% RH and maintained in IEC medium (32). For cell culture under non-polarized conditions (2D), IECs were seeded in 12-well or 24-well plates at a seeding density of 2 × 10^5^ or 2 × 10^4^ cells, respectively, and grown to confluence. For cell culture under polarized conditions (3D), IECs were seeded at a cell density of 2 × 10^5^ cells/mL on 0.4 µm pore size transwell cell-culture inserts (Costar). Cells were allowed to grow for 21 days to attain polarization. The cell-culture medium was changed every 3 days, and transepithelial resistance was measured (EVOM, World Precision instruments) to determine the establishment of epithelial barrier integrity. IECs were stimulated with 500 U/mL IFN-β (19) or 20 ng/mL IFN-λ_2_ (Peprotech) in cell-culture medium.

### Isolation and Culture of Primary Cells

For isolation of primary intestinal epithelial cells, small intestinal tissue was harvested and cut into 3–4 cm pieces. The tissue-associated fat tissue was removed using forceps, and the intestine was turned inside out. The inverted tissue was mounted on an inoculation loop, incubated for 10 min in 30 mM EDTA at 37°C and subjected to centrifugal force with a biovortexer (Sigma) using 10–12 pulses with 1–2 s duration. Epithelial cell fragments were separated from contaminating lymphoid and myeloid single cells by threefold sedimentation at 1 × *g* for 20 min at 4°C leading to a final purity of E-cadherin positive epithelial cells of 85–90% (35). Bone marrow-derived dendritic cells (BMDCs) were obtained from female C57BL/6 WT mice by flushing the bone marrow from the cavities of femurs and tibiae. Erythrocytes were depleted with ACK lysis buffer (Thermo Fisher Scientific), and the cells were plated in 12-well cell-culture plates at a seeding density of 1 × 10^6^ cells/mL in the presence of Flt3L at 100 ng/mL (PeproTech, Rocky Hill, NJ, USA) in complete medium (RPMI supplemented with 10% heat-inactivated fetal bovine serum, 1% penicillin/streptomycin, 1% glutamine, and 50 µg/mL gentamicin). Cultures were replenished with fresh medium every other day and stimulated at day 7. Primary alveolar epithelial cells were isolated using a modified protocol previously established (36). Briefly, the trachea of the anesthetized and exsanguinated mice was exposed and the lungs were perfused with 10–20 mL sterile PBS buffer until they were free of blood. 1 mL of dispase (BD Biosciences) was flushed into the lungs *via* the trachea. The lungs were removed and placed in a cell-culture dish containing an additional 1 mL of dispase and were cut into small pieces. They were then transferred to a 15 mL Falcon and incubated for 45 min at 37°C with gentle shaking. The crude cell suspension was passed through a sterile 70 µm strainer, and the resulting cell suspension was centrifuged at 1,500 rpm for 5 min. The pellet obtained was incubated in 5 mL of ACK buffer for erythrocyte depletion for 5 min and subsequently subjected to another round of centrifugation at 1,500 rpm for 5 min. Cells were stained with Epcam-PE (eBioscience) and magnetically sorted (MACS anti-PE Microbeads, Miltenyi Biotec GmbH) to obtain a highly enriched population of epithelial cells. Cells were plated in 12-well cell-culture plates at a seeding density of 1 × 10^6^ cells/mL and stimulated after 5 days in culture.

### Gene Expression Analysis

RNA from cell-culture experiments was isolated using the RNeasy mini kit (Qiagen) based on silica membrane containing centrifugation columns following the manufacturer’s instructions. Total RNA from primary epithelial cells was isolated by guanidinium thiocyanate-phenol-chloroform extraction using Trizol LS reagent (Life Technologies) according to the manufacturer’s instructions. 1–2 µg RNA was reversely transcribed into cDNA using the RevertAid RT Kit (Thermo Fisher Scientific). Newly synthesized cDNA was subjected to quantitative real-time PCR analysis in a total volume of 20 µl, using the SYBR Green PCR Kit (BioRad) in combination with a LightCycler 480 II (Roche). The expression level of the house-keeping gene *β-actin* in IEC10 cells was unaffected by IFN-β or IFN-λ_2_ stimulation (Figure S2A in Supplementary Material). Changes in gene expression were calculated relative to the endogenous control *β-actin* using the formula 2^−ΔCt^. Experiments demonstrated no influence of IFN stimulation on the *β-actin* mRNA expression level (data not shown). The values obtained for individual genes after stimulation with IFN-β or IFN-λ_2_ were subsequently divided by the mean values found in untreated cells (PBS). Gene expression values are presented as fold induction over the unstimulated control. Statistical analysis was performed using a (non-parametric) one-way analysis of variance (ANOVA) test with Tukey’s post test, and the data are presented as mean ± SEM. The values obtained for *in vivo* gene expression were normalized to the endogenous control β-actin, statistically analyzed by the Mann–Whitney *U* test and are represented as mean ± SEM from two to three independent experiments. Murine PCR primers for *β-actin* (forward primer, 5′-TGG AAT CCT GTG GCA TCC ATG AAA C-3′ and reverse primer, 5′-TAA AAC GCA GCT CAG TAA CAG TCC G-3′), *Usp18* (forward primer, 5′-CAT CCT CCA GGG TTT TCA GA-3′ and reverse primer, 5′-AAG GAC CAG ATC ACG GAC AC-3′), *Ifi44* (forward primer, 5′-AAC TGA CTG CTC GCA ATA ATG T-3′ and reverse primer, 5′-GTA ACA CAG CAA TGC CTC TTG T-3′), *Ifit1* (forward primer, 5′-TGT TGA AGC AGA AGC ACA CA-3′ and reverse primer, 5′-TCT ACG CGA TGT TTC CTA CG-3′), *Mmp7* (forward primer, 5′-TAG GCG GAG ATG CTC ACT TT-3′ and reverse primer, 5′-TTC TGA ATG CCT GCA ATG TC-3′), *Serpinb1a* (forward primer, 5′-GCT GCT ACA GGA GGC ATT GC-3′ and reverse primer, 5′-CGG ATG GTC CAC TGT GAA TTC-3′), *Csprs* (forward primer, 5′-AGA GAG GCA GAG GGA CTG AG-3′ and reverse primer, 5′-GGC TTG GCT CCT GAA CAC TT-3′), *IL28R* (forward primer, 5′-CCC TGT TTC CTG ACA CTC CC-3′ and reverse primer, 5′-TCA GAA AAG TCC AGT GCC CG-3′), *IL10R* (forward primer, 5′-TCT CTT CCA CAG CAC C-3′ and reverse primer, 5′-GAA CAC CTC GCC CTC C-3′), *Ifnar1* (forward primer, 5′-CTG GTC TGT GAG CTG TAC TT-3′ and reverse primer, 5′-TCC CCG CAG TAT TGA TGA GT-3′), *Ifnar2* (forward primer, 5′-CTA TCG TAA TGC TGA AAC GG-3′ and reverse primer, 5′-CGT AAT TCC ACA GTC TCT TCT-3′).

### Microarray Analysis

**Figure 1:**
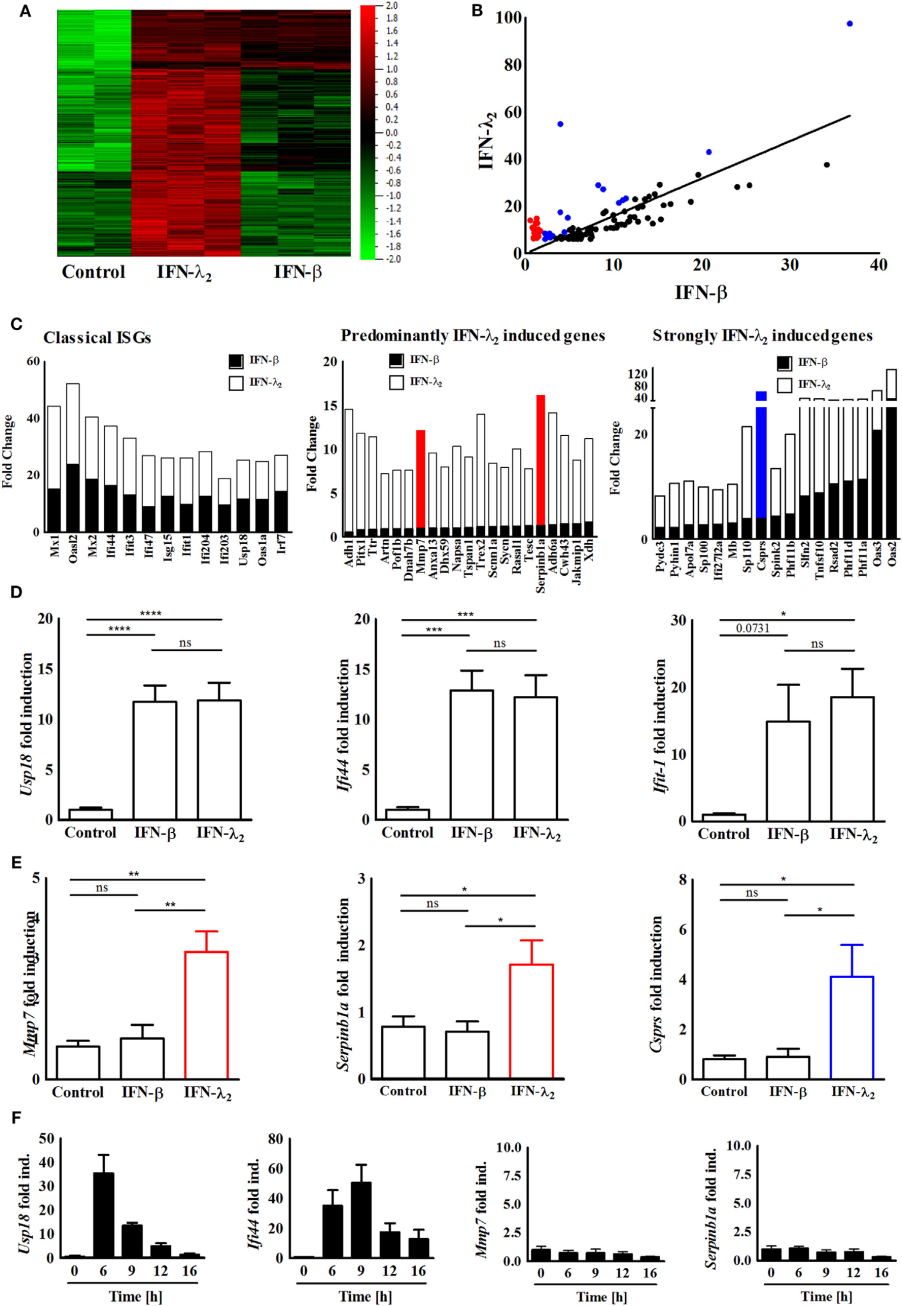
Identification and confirmation of a predominantly interferon (IFN)-λ_2_-induced gene expression profile. **(A)** Heatmap of the genes expressed by IEC10 cells cultured on transwell filter inserts and left untreated (PBS) or exposed to IFN-λ_2_ (20 ng/mL) or IFN-β (500 U/mL) for 9 h. Data were obtained using a global gene expression array. Multigroup comparison was carried out at *p* = 0.003, *q* = 0.05. **(B)** Selective analysis of the top 100 genes induced by IFN-λ_2_ as identified by fold increase over unstimulated control. Correlation graph showing the fold change of these 100 genes in respect to their induction by IFN-λ_2_ (20 ng/mL) versus IFN-β (500 U/mL) 9 h after stimulation. Red labeled dots illustrate a subgroup of genes that is predominantly induced by IFN-λ2; blue dots illustrate a subgroup of genes strongly induced by IFN-λ2 (for definition see Section “Materials and Methods”). **(C)** Graphical representation showing the fold change analysis of different gene subgroups [“classical antiviral IFN-stimulated genes (ISGs),” “predominantly IFN-λ_2_-induced genes,” “strongly IFN-λ_2_-induced genes”]. **(D,E)** Quantitative RT-PCR for **(D)** the prototypical ISGs *Usp18, Ifi44*, and *Ifit1*, **(E)** the predominantly IFN-λ_2_-induced ISGs *Mmp7, Serpinb1a*, and the strongly IFN-λ_2_-induced gene *Csprs* performed on total RNA isolated from IEC10 cells grown on transwell inserts and stimulated for 9 h with IFN-λ_2_ (20 ng/mL) or IFN-β (500 U/mL). The results are represented as mean ± SEM values from two to three independent experiments and are normalized to the values obtained for the housekeeping gene *β-actin*. Statistical significance was calculated using the one-way analysis of variance (with Tukey’s posttest). **(F)** Quantitative RT-PCR for the prototypical ISGs *Usp18* and *Ifi44* as well as the predominantly IFN-λ_2_-induced ISGs *Mmp7* and *Serpinb1a* performed on total RNA isolated from IEC10 cells grown on transwell filter inserts and stimulated with IFN-β (500 U/mL) for the indicated time period. The results represent the mean ± SEM values from two to three independent experiments and are normalized to the values obtained for the housekeeping gene *β-actin*.

Microarray analysis was performed in triplicates on 3D-grown unstimulated or IFN-stimulated IECs. RNA was extracted using the RNeasy Mini Kit (Qiagen) according to the manufacturer’s protocol. Microarray data used or referred to in this publication were generated by the Research Core Unit Transcriptomics of Hannover Medical School. Synthesis of Cy3-labeled cRNA was performed with the Quick Amp Labeling kit, one color (Agilent Technologies) according to the manufacturer’s recommendations. cRNA fragmentation, hybridization, and washing steps were also carried out exactly as recommended: “One-Color Microarray-Based Gene Expression Analysis Protocol V5.7.” Microarray analysis was performed using Whole Mouse Genome Oligo Microarray GPL11202 (Agilent Technologies). Slides were scanned on the Agilent Micro Array Scanner G2565CA (pixel resolution 5 µm, bit depth 20). Data extraction was performed with the “Feature Extraction Software V10.7.3.1” by using the recommended default extraction protocol file: “GE1_107_Sep09.xml.” Measurements of on-chip replicates were averaged using the geometric mean of processed intensity values of the green channel, “gProcessedSignal” (gPS) to retrieve one resulting value per unique non-control probe. Single features were excluded from averaging, if they (i) were manually flagged, (ii) were identified as outliers by the feature extraction software, (iii) lie outside the interval of “1.42 × interquartile range” regarding the normalized gPS distribution of the respective on-chip replicate population, or, (iv) showed a coefficient of variation of pixel intensities per Feature that exceeded 0.5. Averaged gPS values were normalized by global linear scaling. For this approach, all gPS values of one sample were multiplied by an array-specific scaling factor. This factor was calculated by dividing a “reference 75th Percentile value” (set as 1,500 for the whole series) by the 75th Percentile value of the particular Microarray to be scaled (“Array I” in the formula shown below). Accordingly, normalized gPS values for all samples (microarray data sets) were calculated by the following formula: normalized gPSArray i = gPSArray i × (1,500/75th PercentileArray i). A lower intensity threshold (surrogate value) was defined based on intensity distribution of negative control features. This value was fixed at 15 normalized gPS units. All measurements that fell below this intensity cutoff were substituted by the respective surrogate value of 15. The hierarchical clustering heatmap was generated using Qlucore Omics explorer (multigroup analysis: *p*-value = 0.003; *q*-value = 0.05; two-group analysis: *p*-value = 0.001; *q*-value = 0.05, fold change cutoff = 2). The group definitions for the IFN-induced genes (Figure 1C) were as follows: “predominantly IFN-λ_2_-induced gene”: fold increase by IFN-λ_2_ over control/fold increase by IFN-β over control >4.5 and fold increase by IFN-β over control <2; “strong IFN-λ_2_-induced gene”: fold increase by IFN-λ_2_ over control/fold increase by IFN-β over control >2 and fold increase by IFN-β over control >2: “Classical ISGs” were defined by their designation in the literature. Cluster of orthologous group analysis was performed using the PANTHER software.^2^ Expression array data are available through GEO Series accession number GSE91382.

### Statistical Analysis

The one-way ANOVA test (with Tukey’s posttest) and the Mann–Whitney *U* test were employed for statistical analysis of quantitative RT-PCR results. The GraphPad Prism Software 7.00 was used for statistical evaluation.

## Results

### IFN-λ_2_ Induces a Unique Transcriptional Profile in Polarized IECs

The recently described intestinal epithelial IEC10 cells exhibit many properties of the natural epithelium. They respond to both type I and type III IFNs and generate a robust antiviral state making them an ideal model to study IFN-induced gene expression (32). IEC10 cells were grown to confluency on transwell cell-culture inserts and left untreated or stimulated with IFN-β (500 U/mL) or IFN-λ_2_ (20 ng/mL) for 9 h. The selected IFN concentrations induced a submaximal stimulatory response (approximately 90% of the maximal Mx2 gene induction) in IEC10 cells for both cytokines as recently reported (32). Similar IFN concentrations have also been used in other comparative studies (9, 12, 20, 24–30). The stimulation time (9 h) was selected based on the kinetic of ISG (Mx2) induction following IFN-β and/or IFN-λ_2_ exposure and allowed a stable gene induction for both cytokines (Figure S1B in Supplementary Material). Total RNA was isolated and subjected to transcriptome analysis. Normalization and multigroup analysis (ANOVA) revealed a total of 2,465 significantly differentially regulated genes (*q*-value = 0.05, *p*-value = 0.003). Figure 1A illustrates the genes significantly induced by IFN-β and/or IFN-λ. In a second approach, we subjected the genes through a two-group analysis (filtering criteria: *q*-value = 0.0499, *p*-value = 0.001, fold change cutoff = 2) and observed that 349 genes were highly expressed after stimulation with IFN-λ_2_ but not IFN-β (Figure S2A in Supplementary Material). In a third approach, the top 100 (fold over control) IFN-λ_2_-induced genes were selected (Table 1) and examined in a correlation analysis for their induction by IFN-β versus IFN-λ_2_ revealing a majority of classical ISGs including the prototypical antiviral genes *Ifi44* and *Ifit1* as presented in Figures 1B,C (left panel). Among these genes, also a group of genes predominantly induced by IFN-λ_2_ and a group of genes strongly induced by IFN-λ_2_ was identified (Figure 1B labeled in red and blue, respectively, and Figure 1C middle and right panel). These genes were found to be mainly involved in cellular and metabolic processes and cellular responses to stimuli such as innate host defense, substrate transport and ion homeostasis (Figures S2B,C in Supplementary Material). Two predominantly IFN-λ_2_-induced genes, *Mmp7* and *Serpinb1a*, one strongly IFN-λ_2_-induced gene, *Csprs*, as well as the classical antiviral ISGs *Usp18, Ifi44*, and *Ifit1* were randomly selected and their transcriptional profile upon stimulation with type I or type III IFN for 9 h was confirmed by quantitative RT-PCR (Figures 1D,E). IFN-β was unable to induce expression of the predominantly IFN-λ_2_-induced genes *Mmp7* and *Serpinb1a* also at other time points (Figure 1F; Figure S3 in Supplementary Material).

**Table 1:** Top 100 genes induced by IFN-λ2.

Accession ID	Description	Gene name	IFN-λ_2_ fold change	IFN-β fold change	IFN-λ2/IFN-β
NM_145227	*Mus musculus* 2′-5′ oligoadenylate synthetase 2 (Oas2), mRNA [NM_145227]	Oas2	97.48	36.70	2.66
NM_033616	*M. musculus* component of Sp100-rs (Csprs), mRNA [NM_033616]	Csprs	54.87	3.97	13.82
NM_145226	*M. musculus* 2′-5′ oligoadenylate synthetase 3 (Oas3), mRNA [NM_145226]	Oas3	43.04	20.77	2.07
NM_001139519	*M. musculus* Z-DNA binding protein 1 (Zbp1), transcript variant 2, mRNA [NM_001139519]	Zbp1	37.57	34.11	1.10
NM_030150	*M. musculus* DEXH (Asp–Glu–X-His) box polypeptide 58 (Dhx58), mRNA [NM_030150]	Dhx58	33.28	19.56	1.70
NM_010846	*M. musculus* myxovirus (influenza virus) resistance 1 (Mx1), mRNA [NM_010846]	Mx1	29.16	15.19	1.92
NM_011408	*M. musculus* schlafen 2 (Slfn2), mRNA [NM_011408]	Slfn2	28.93	8.24	3.51
NM_001289492	*M. musculus* guanylate binding protein 3 (Gbp3), transcript variant 1, mRNA [NM_001289492]	Gbp3	28.85	25.35	1.14
NM_011854	*M. musculus* 2′-5′ oligoadenylate synthetase-like 2 (Oasl2), mRNA [NM_011854]	Oasl2	28.19	23.97	1.18
NM_009425	*M. musculus* tumor necrosis factor (ligand) superfamily, member 10 (Tnfsf10), mRNA [NM_009425]	Tnfsf10	27.23	8.80	3.09
NM_001168660	*M. musculus* apolipoprotein L 9b (Apol9b), transcript variant 1, mRNA [NM_001168660]	Apol9b	25.14	14.63	1.72
NM_173786	*M. musculus* apolipoprotein L 9a (Apol9a), transcript variant 1, mRNA [NM_173786]	Apol9a	24.11	13.99	1.72
NM_172603	*M. musculus* PHD finger protein 11A (Phf11a), mRNA [NM_172603]	Phf11a	23.35	11.38	2.05
NM_010821	*M. musculus* macrophage expressed gene 1 (Mpeg1), mRNA [NM_010821]	Mpeg1	22.94	13.51	1.70
NM_001146275	*M. musculus* interferon-inducible GTPase 1 (Iigp1), transcript variant 2, mRNA [NM_001146275]	Iigp1	22.93	12.41	1.85
NM_199015	*M. musculus* PHD finger protein 11D (Phf11d), mRNA [NM_199015]	Phf11d	22.59	11.04	2.05
NM_013606	*M. musculus* myxovirus (influenza virus) resistance 2 (Mx2), transcript variant 1, mRNA [NM_013606]	Mx2	21.84	18.72	1.17
NM_021384	*M. musculus* radical *S*-adenosyl methionine domain containing 2 (Rsad2), mRNA [NM_021384]	Rsad2	21.38	10.58	2.02
NM_133871	*M. musculus* interferon (IFN)-induced protein 44 (Ifi44), mRNA [NM_133871]	Ifi44	20.93	16.42	1.27
NM_009099	*M. musculus* tripartite motif-containing 30A (Trim30a), mRNA [NM_009099]	Trim30a	20.39	15.62	1.31
NM_010501	*M. musculus* IFN-induced protein with tetratricopeptide repeats 3 (Ifit3), mRNA [NM_010501]	Ifit3	19.90	13.26	1.50
NM_199146	*M. musculus* tripartite motif-containing 30D (Trim30d), transcript variant 1, mRNA [NM_199146]	Trim30d	19.29	12.74	1.52
NM_001145164	*M. musculus* T cell-specific GTPase 2 (Tgtp2), mRNA [NM_001145164]	Tgtp2	17.80	11.22	1.59
NM_001271676	*M. musculus* IFN-λ-inducible protein 47 (Ifi47), transcript variant 2, mRNA [NM_001271676]	Ifi47	17.74	9.09	1.95
NM_175397	*M. musculus* Sp110 nuclear body protein (Sp110), transcript variant 1, mRNA [NM_175397]	Sp110	17.45	3.95	4.42
NM_011579	*M. musculus* T cell-specific GTPase 1 (Tgtp1), mRNA [NM_011579]	Tgtp1	16.98	8.83	1.92
NM_008331	*M. musculus* IFN-induced protein with tetratricopeptide repeats 1 (Ifit1), mRNA [NM_008331]	Ifit1	16.27	9.94	1.64
ENSMUST00000 102642	Ubiquitin-conjugating enzyme E2L 6 [source:MGI Symbol;Acc:MGI: 1914500] [ENSMUST00000102642]	Ube2l6	15.38	12.34	1.25
NM_001037713	*M. musculus* XIAP-associated factor 1 (Xaf1), transcript variant 1, mRNA [NM_001037713]	Xaf1	15.35	11.86	1.29
NM_001164327	*M. musculus* PHD finger protein 11B (Phf11b), mRNA [NM_001164327]	Phf11b	15.13	4.81	3.15
XM_006497295	PREDICTED: *M. musculus* IFN-activated gene 204 (Ifi204), transcript variant X1, mRNA [XM_006497295]	Ifi204	14.98	13.86	1.08
NM_025429	*M. musculus* serine (or cysteine) peptidase inhibitor, clade B, member 1a (Serpinb1a), mRNA [NM_025429]	Serpinb1a	14.83	1.29	11.51
NM_001045481	*M. musculus* IFN-activated gene 203 (Ifi203), transcript variant 1, mRNA [NM_001045481]	Ifi203	14.38	15.34	0.94
NM_020557	*M. musculus* cytidine monophosphate (UMP-CMP) kinase 2, mitochondrial (Cmpk2), mRNA [NM_020557]	Cmpk2	14.15	10.05	1.41
NM_007409	*M. musculus* alcohol dehydrogenase 1 (class I) (Adh1), mRNA [NM_007409]	Adh1	13.97	0.55	25.50
NM_011909	*M. musculus* ubiquitin-specific peptidase 18 (Usp18), mRNA [NM_011909]	Usp18	13.66	11.75	1.16
NM_015783	*M. musculus* ISG15 ubiquitin-like modifier (Isg15), mRNA [NM_015783]	Isg15	13.41	12.75	1.05
NM_145211	*M. musculus* 2′-5′ oligoadenylate synthetase 1A (Oas1a), mRNA [NM_145211]	Oas1a	13.37	11.59	1.15
NM_011907	*M. musculus* three prime repair exonuclease 2 (Trex2), mRNA [NM_011907]	Trex2	12.85	1.15	11.16
NM_026945	*M. musculus* alcohol dehydrogenase 6A (class V) (Adh6a), mRNA [NM_026945]	Adh6a	12.79	1.37	9.37
NM_016850	*M. musculus* interferon regulatory factor 7 (Irf7), transcript variant 1, mRNA [NM_016850]	Irf7	12.58	14.41	0.87
NM_001039530	*M. musculus* poly (ADP-ribose) polymerase family, member 14 (Parp14), mRNA [NM_001039530]	Parp14	12.29	10.84	1.13
NM_001033450	*M. musculus* myeloid cell nuclear differentiation antigen (Mnda), mRNA [NM_001033450]	Mnda	12.14	11.22	1.08
NM_145211	*M. musculus* 2′-5′ Oas1a, mRNA [NM_145211]	Oas1a	12.10	10.42	1.16
NM_010810	*M. musculus* matrix metallopeptidase 7 (Mmp7), mRNA [NM_010810]	Mmp7	11.17	0.97	11.57
NM_011097	*M. musculus* paired-like homeodomain transcription factor 1 (Pitx1), mRNA [NM_011097]	Pitx1	11.00	0.80	13.70
NM_023386	*M. musculus* receptor transporter protein 4 (Rtp4), mRNA [NM_023386]	Rtp4	10.93	10.34	1.06
NM_010260	*M. musculus* guanylate binding protein 2 (Gbp2), mRNA [NM_010260]	Gbp2	10.82	9.66	1.12
NM_007986	*M. musculus* fibroblast activation protein (Fap), mRNA [NM_007986]	Fap	10.68	5.36	1.99
NM 028967	*M. musculus* basic leucine zipper transcription factor, ATF-like 2 (Batf2), mRNA [NM_028967]	Batf2	10.66	9.97	1.07
NM_013697	*M. musculus* transthyretin (Ttr), mRNA [NM_013697]	Ttr	10.58	0.86	12.33
NM_145153	*M. musculus* 2′-5′ oligoadenylate synthetase 1F (Oas1f), mRNA [NM_145153]	Oas1f	10.56	9.16	1.15
NM_001146007	*M. musculus* tripartite motif-containing 12C (Trim12c), transcript variant 1, mRNA [NM_001146007]	Trim12c	10.50	7.49	1.40
NM_019440	*M. musculus* immunity-related GTPase family M member 2 (Irgm2), mRNA [NM_019440]	Irgm2	10.22	7.98	1.28
NM_181323	*M. musculus* cell wall biogenesis 43 C-terminal homolog (*S. cerevisiae*) (Cwh43), mRNA [NM_181323]	Cwh43	10.08	1.49	6.76
NM_194336	*M. musculus* guanylate binding protein 6 (Gbp6), mRNA [NM_194336]	Gbp6	9.99	6.01	1.66
NM_001170853	*M. musculus* myeloid nuclear differentiation antigen like (Mndal), mRNA [NM_001170853]	Mndal	9.94	7.07	1.41
NM_001256005	*M. musculus* guanylate binding protein 4 (Gbp4), transcript variant 1, mRNA [NM_001256005]	Gbp4	9.93	5.02	1.98
NM_011723	*M. musculus* xanthine dehydrogenase (Xdh), mRNA [NM_011723]	Xdh	9.49	1.71	5.54
NM_008437	*M. musculus* napsin A aspartic peptidase (Napsa), mRNA [NM_008437]	Napsa	9.31	1.02	9.13
NM_183284	*M. musculus* serine peptidase inhibitor, Kazal type 2 (Spink2), transcript variant 2, mRNA [NM_183284]	Spink2	9.00	4.41	2.04
NM_013832	*M. musculus* RAS protein activator like 1 (GAP1 like) (Rasal1), transcript variant 1, mRNA [NM_013832]	Rasal1	8.79	1.23	7.15
NM_027211	*M. musculus* annexin A13 (Anxa13), mRNA [NM_027211]	Anxa13	8.58	1.00	8.58
NM_145209	*M. musculus* 2′-5′ oligoadenylate synthetase-like 1 (Oasl1), mRNA [NM_145209]	Oasl1	8.48	5.36	1.58
NM_008505	*M. musculus* LIM domain only 2 (Lmo2), transcript variant 1, mRNA [NM_008505]	Lmo2	8.40	6.16	1.36
NM_175026	*M. musculus* pyrin and HIN domain family, member 1 (Pyhin1), mRNA [NM_175026]	Pyhin1	8.38	2.22	3.77
NM_029419	*M. musculus* apolipoprotein L 7a (Apol7a), transcript variant 1, mRNA [NM_029419]	Apol7a	8.36	2.73	3.06
NM_023141	*M. musculus* torsin family 3, member A (Tor3a), mRNA [NM_023141]	Tor3a	8.32	7.42	1.12
NM_008326	*M. musculus* immunity-related GTPase family M member 1 (Irgm1), mRNA [NM_008326]	Irgm1	8.16	6.84	1.19
NM_011852	*M. musculus* 2′-5′ oligoadenylate synthetase 1G (Oas1g), mRNA [NM_011852]	Oas1g	8.11	5.56	1.46
NM_145545	*M. musculus* guanylate binding protein 7 (Gbp7), transcript variant 1, mRNA [NM_145545]	Gbp7	8.11	6.28	1.29
NM_010708	*M. musculus* lectin, galactose binding, soluble 9 (Lgals9), transcript variant 1, mRNA [NM_010708]	Lgals9	8.06	6.89	1.17
NM_133681	*M. musculus* tetraspanin 1 (Tspan1), mRNA [NM_133681]	Tspan1	8.05	1.07	7.56
NM_010426	*M. musculus* forkhead box F1 (Foxf1), mRNA [NM_010426]	Foxf1	7.67	6.22	1.23
NM_013593	*M. musculus* myoglobin (Mb), transcript variant 2, mRNA [NM_013593]	Mb	7.33	3.09	2.37
NM_178394	*M. musculus* janus kinase and microtubule interacting protein 1 (Jakmip1), mRNA [NM_178394]	Jakmip1	7.24	1.49	4.85
NM_013673	*M. musculus* nuclear antigen Sp100 (Sp100), mRNA [NM_013673]	Sp100	7.23	2.74	2.64
NM_011324	*M. musculus* sodium channel, non-voltage-gated 1 alpha (Scnn1a), mRNA [NM_011324]	Scnn1a	7.22	1.18	6.10
NM_145226	*M. musculus* 2′-5′ oligoadenylate synthetase 3 (Oas4), mRNA [NM_145226]	Oas4	7.04	3.98	1.77
NM_001139519	*M. musculus* Z-DNA binding protein 2 (Zbp2), transcript variant 2, mRNA [NM_001139519]	Zbp2	6.99	6.35	1.10
NM_030150	*M. musculus* DEXH (Asp–Glu–X-His) box polypeptide 58 (Dhx58), mRNA [NM_030150]	Dhx59	6.98	1.01	6.93
NM_025378	*M. musculus* IFN-induced transmembrane protein 3 (Ifitm3), mRNA [NM_025378]	Ifitm3	6.94	7.55	0.92
NM_197944	*M. musculus* hematopoietic SH2 domain containing (Hsh2d), mRNA [NM_197944]	Hsh2d	6.72	4.40	1.53
NM_001160386	*M. musculus* dynein, axonemal, heavy chain 7B (Dnah7b), mRNA [NM_001160386]	Dnah7b	6.72	0.92	7.29
NM_026716	*M. musculus* syncollin (Sycn), mRNA [NM_026716]	Sycn	6.71	1.21	5.53
NM_023835	*M. musculus* tripartite motif-containing 12A (Trim12a), mRNA [NM_023835]	Trim12a	6.70	5.83	1.15
NM_181579	*M. musculus* premature ovarian failure 1B (Pof1b), mRNA [NM_181579]	Pof1b	6.68	0.92	7.26
NM_029803	*M. musculus* IFN, alpha-inducible protein 27-like 2A (Ifi27l2a), transcript variant 1, mRNA [NM_029803]	Ifi27l2a	6.65	2.82	2.36
NM_021344	*M. musculus* tescalcin (Tesc), mRNA [NM_021344]	Tesc	6.51	1.28	5.09
NM_181728	*M. musculus* ADP-ribosyltransferase 3 (Art3), mRNA [NM_181728]	Art3	6.45	5.17	1.25
NM_001284192	*M. musculus* artemin (Artn), transcript variant 2, mRNA [NM_001284192]	Artn	6.29	0.91	6.91
NM_029000	*M. musculus* GTPase, very large IFN-inducible 1 (Gvin1), transcript variant 1, mRNA [NM_029000]	Gvin1	6.16	5.74	1.07
NM_013585	*M. musculus* proteasome (prosome, macropain) subunit, beta type 9 (large multifunctional peptidase 2) (Psmb9), mRNA [NM_013585]	Psmb9	6.16	3.51	1.76
NM_001146007	*M. musculus* tripartite motif-containing 12C (Trim12c), transcript variant 1, mRNA [NM_001146007]	Trim12c	6.11	5.41	1.13
NM_023141	*M. musculus* torsin family 3, member A (Tor3a), mRNA [NM_023141]	Tor3a	6.11	5.96	1.03
NR_030671	*M. musculus* expressed sequence AW011738 (AW011738), long non-coding RNA [NR_030671]	AW01173 8	6.11	7.36	0.83
NM_021274	*M. musculus* chemokine (C–X–C motif) ligand 10 (Cxcl10), mRNA [NM_021274]	Cxcl10	6.10	4.89	1.25
NM_001025208	*M. musculus* MHC class I family member (LOC547349), mRNA [NM_001025208]	LOC5473 49	6.10	4.08	1.49
NM_030253	*M. musculus* poly (ADP-ribose) polymerase family, member 9 (Parp9), mRNA [NM_030253]	Parp9	6.04	6.28	0.96
NM_001162938	*M. musculus* pyrin domain containing 3 (Pydc3), mRNA [NM_001162938]	Pydc3	6.03	2.21	2.72

### Expression of Predominantly IFN-λ_2_-Induced Genes Requires Epithelial Cell Polarization

**Figure 2:**
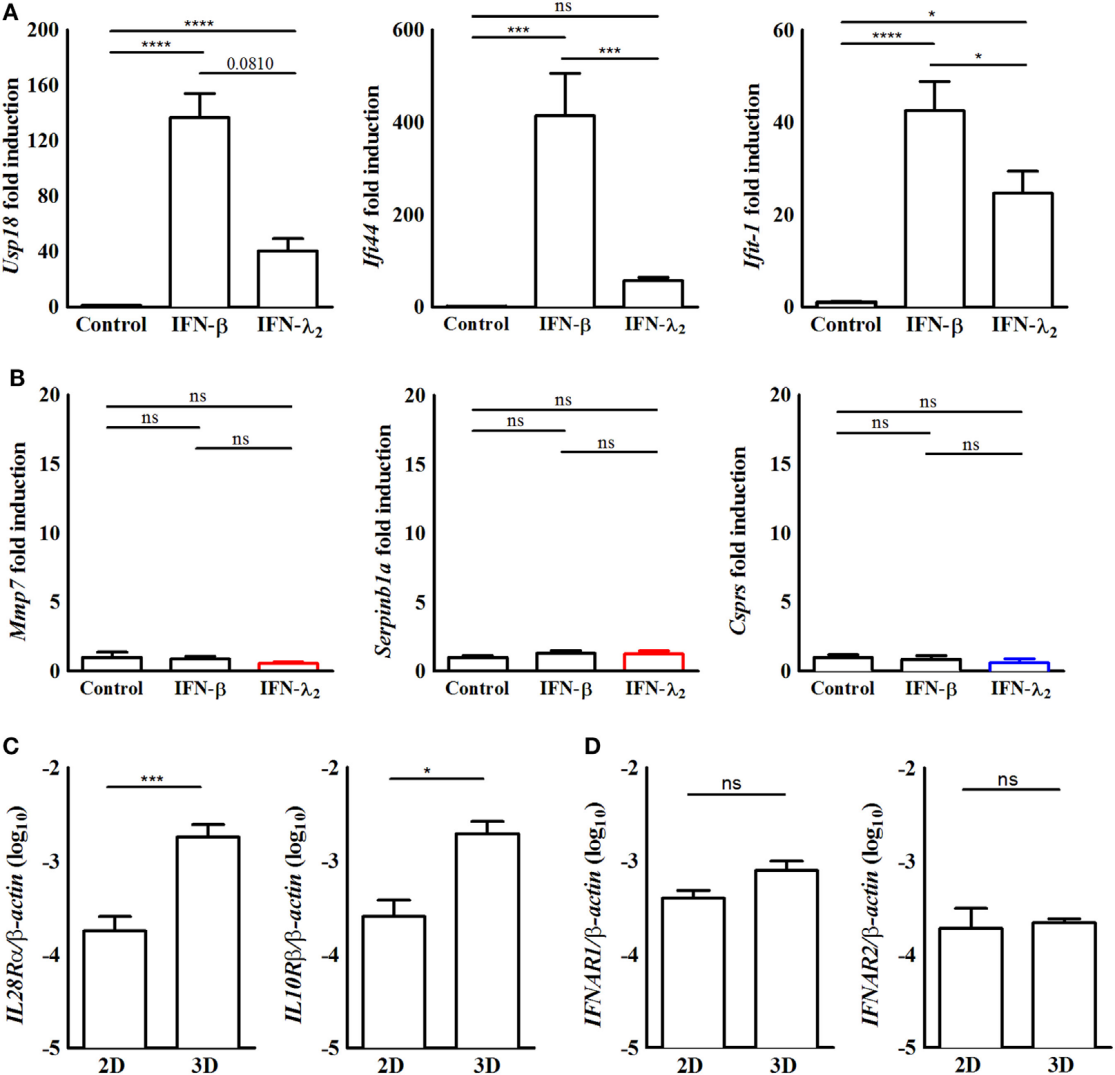
The induction of predominantly interferon (IFN)-λ_2_-induced genes requires epithelial polarization. **(A,B)** Quantitative RT-PCR for **(A)** the prototypical IFN-stimulated genes (ISGs) *Usp18*, I*fi44*, and *Ifit1* or **(B)** the predominantly IFN-λ-induced ISGs *Mmp7, Serpinb1a*, and *Csprs* performed on total RNA isolated from IEC10 cells grown in conventional flat bottom 12-well tissue culture plates under 2D conditions and stimulated for 9 h with IFN-λ_2_ (20 ng/mL) or IFN-β (500 U/mL). The results represent the mean ± SEM values from two independent experiments and are normalized to the values obtained for the housekeeping gene *β-actin*. Statistical significance was calculated using a one-way analysis of variance (with Tukey’s posttest). **(C,D)** Quantitative RT-PCR for **(C)** IL-28Rα and IL-10 receptor (IL-10R)β and **(D)** IFNAR1 and IFNAR2 performed on total RNA isolated from unstimulated IEC10 cells grown either on conventional flat bottom tissue culture plates (2D) or transwell inserts (3D). The results are normalized to the values obtained for β-actin and are represented as mean ± SEM values from two independent experiments performed in quadruplicates. Statistical significance was calculated using the Mann–Whitney *U* test.

Apical–basolateral polarization represents a key feature of intestinal epithelial cells and is intimately linked to their physiological function such as barrier formation and nutrient absorption. To investigate the influence of cell polarization on IFN-induced gene expression, IEC10 cells were grown on conventional flat bottom culture dishes (2D) and stimulated with IFN-β (500 U/mL) or IFN-λ_2_ (20 ng/mL) for 9 h. RT-PCR confirmed the ability of IFN-β and IFN-λ_2_ to enhance the expression of the prototypical ISGs *Usp18, Ifi44*, and *Ifit1* (Figure 2A). The Usp18, Ifi44, and Ifit1 mRNA levels reached in response to IFN-λ_2_ were less pronounced as compared with under polarized conditions. Due to the lower gene expression levels of unstimulated controls, however, the fold induction was unchanged or even increased (Figure 1D). Notably, IFN-λ_2_ failed to enhance the expression of *Mmp7, Serpinb1a*, and *Csprs* under non-polarizing conditions (Figure 2B). Epithelial polarization might therefore critically influence the qualitative IFN-λ response. In an attempt to understand the underlying mechanism, IEC10 cells grown on flat bottom culture dishes (2D) or transwell inserts (3D) were comparatively examined for the expression levels of the IFN receptor molecules under homeostatic conditions. Epithelial cells displayed significantly increased levels of the IL-28Rα and IL-10Rβ chain expression when they attained polarization as compared with their non-polarized state (Figure 2C) whereas no influence of polarization was noted for the type I IFN receptor IFNAR1 and 2 (Figure 2D) consistent with a recent report (37).

### Cell-Type Specificity of the IFN-λ-Induced Transcriptional Profile

**Figure 3:**
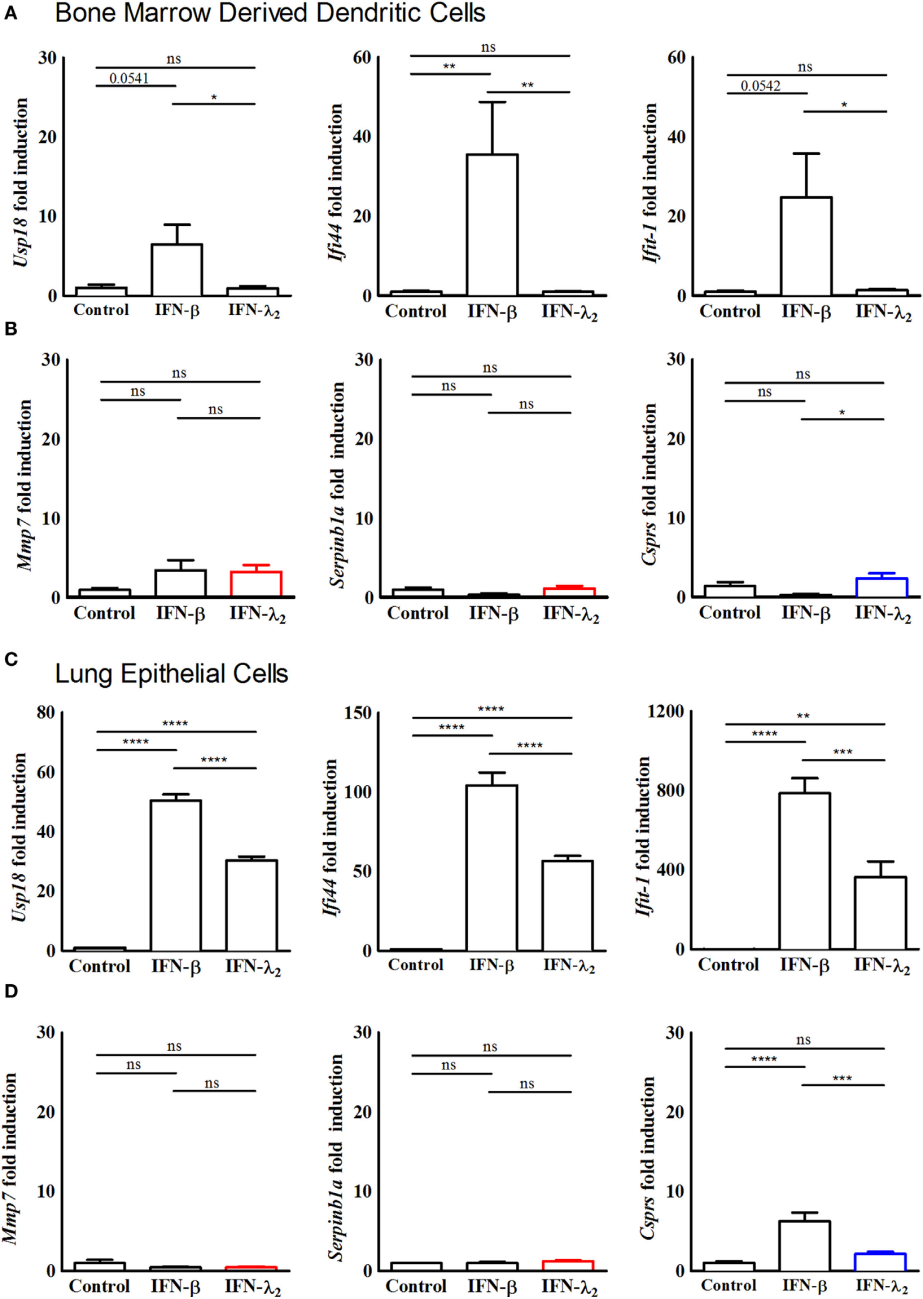
Predominantly interferon (IFN)-λ-induced genes are not induced in bone marrow-derived dendritic cells and primary lung epithelial cells. **(A,B)** Quantitative RT-PCR for **(A)** the prototypical IFN-stimulated genes (ISGs) *Usp18, Ifi44*, and *Ifit1* and **(B)** the predominantly IFN-λ-induced ISGs *Mmp7, Serpinb1a*, and *Csprs* performed on total RNA isolated from bone marrow-derived dendritic cells isolated from 8-week-old female wild-type mice and cultured *in vitro* for 7 days. The dendritic cells were stimulated with Flt3 ligand to initiate maturation, following which they were stimulated with IFN-λ_2_ (20 ng/mL) or IFN-β (500 U/mL) for 9 h. **(C,D)** Quantitative RT-PCR for **(C)** the prototypical ISGs *Usp18, Ifi44*, and *Ifit1* and **(D)** the predominantly IFN-λ-induced ISGs *Mmp7* and *Serpinb1a* and *Csprs* performed on total RNA isolated from primary lung epithelial cells isolated from 8-week-old female wild-type mice and cultured *in vitro* for 5 days before stimulation for 9 h with IFN-λ_2_ (20 ng/mL) or IFN-β (500 U/mL). The results are normalized to β-actin and are represented as mean ± SEM values from two independent experiments performed in quadruplicates. Statistical significance was calculated using a one-way analysis of variance (with Tukey’s posttest).

Dendritic cells were reported to respond to type III IFN (14, 38). Therefore, BMDCs were examined following stimulation with IFN-β (500 U/mL) or IFN-λ_2_ (20 ng/mL) for 9 h by RT-PCR. Expression of the prototypical ISGs *Usp18, Ifi44*, and *Ifit1* was increased following exposure to IFN-β. By contrast, no influence of IFN-λ_2_ on the expression level of *Usp18, Ifi44*, and *Ifit1* (Figure 3A) or the expression level of *Mmp7, Serpinb1a*, and *Csprs* was observed (Figure 3B). To determine if the predominantly IFN-λ_2_-induced gene signature was restricted to the epithelial cells of the intestine, we next analyzed epithelial cells of another important mucosal organ, the lung. Lung epithelial cells have previously been reported to express receptors for both, type I and type III IFNs. Primary lung epithelial cells cultured for 5 days before stimulation were analyzed. Stimulation with IFN-β (500 U/mL) or IFN-λ_2_ (20 ng/mL) for 9 h induced a significant increase of the prototypical ISGs *Usp18, Ifi44*, and *Ifit1* (Figure 3C) but failed to enhance the expression level of *Mmp7* and *Serpinb1a* (Figure 3D). *Csprs* expression was significantly enhanced by IFN-β but not IFN-λ_2_ indicating a more pronounced effect of IFN-λ_2_ on intestinal as compared with lung epithelial cells.

### *In Vivo* Induction of the IFN-λ_2_ Stimulated Gene Signature

**Figure 4:**
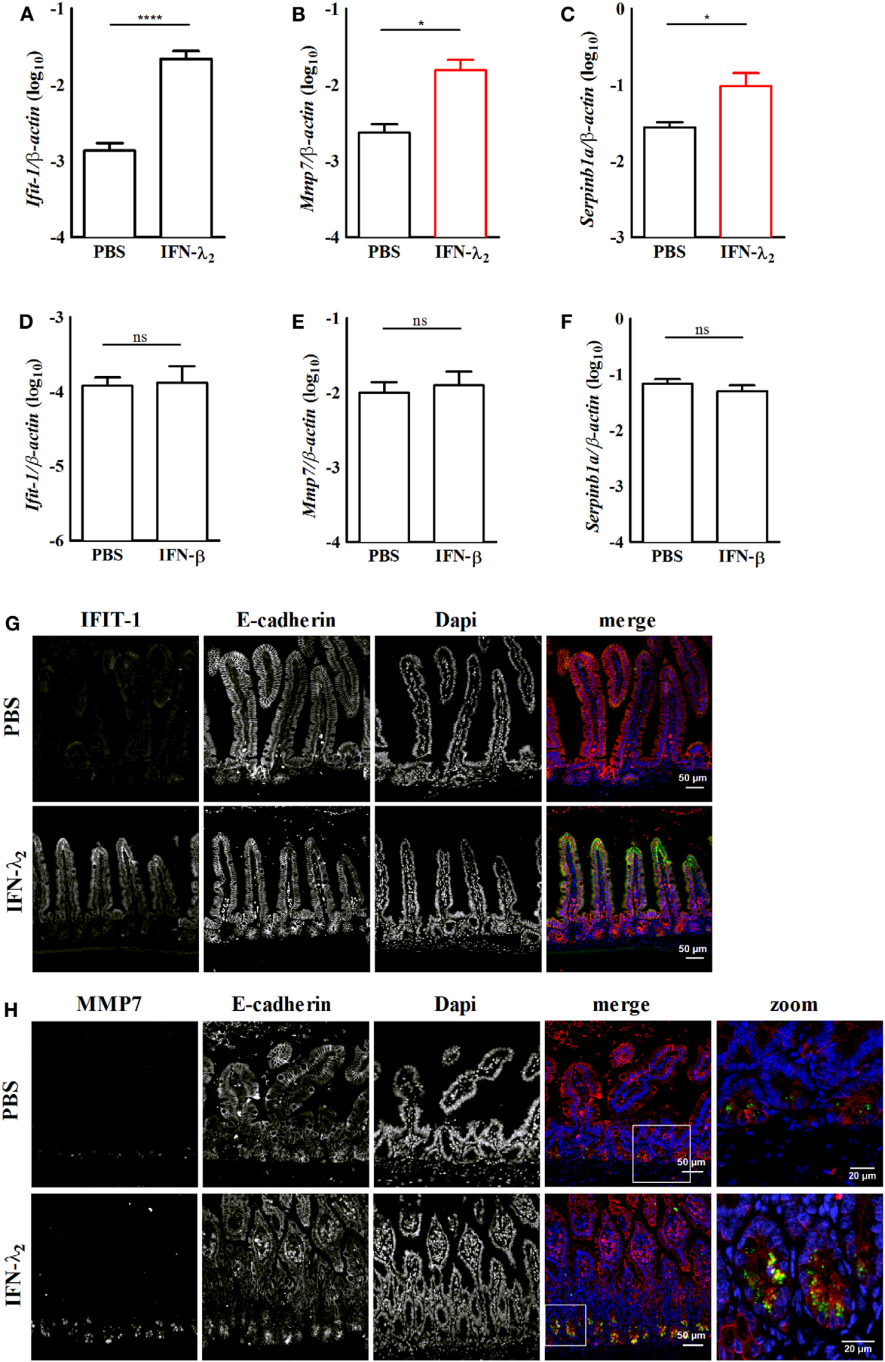
*In vivo* confirmation of the expression of predominantly interferon (IFN)-λ_2_-induced genes in the intestinal epithelium. Primary intestinal epithelial cells were isolated from IFNAR1^−/−^ **(A–C)** or IL28R^−/−^ **(D–F)** female 8-week-old adult mice 9 h after intraperitoneal injection of IFN-λ_2_ [1 µg, **(A)**] or IFN-β [500 U, **(D)**], respectively. Control animals in each group received PBS. Quantitative RT-PCR for the prototypical IFN-stimulated gene (ISG) *Ifit1* [**(A)**, *n* = 12 animals per group; **(D)**, *n* = 8 animals per group], the predominantly IFN-λ_2_-induced ISGs *Mmp7* [**(B)**, *n* = 8 animals per group; **(E)**, 8 animals per group] and *Serpinb1a* [**(C)**, *n* = 12 animals per group; **(F)**, 8 animals per group] performed on total RNA prepared from primary intestinal epithelial cells. Epithelial cells were isolated from PBS or IFN-λ_2_ treated IFNAR1^−/−^ mice or PBS or IFN-β treated IL28R^−/−^ animals. The results are normalized to β-actin and are represented as mean ± SEM from two to three independent experiments. Statistical analysis was performed using the Mann–Whitney *U* test. **(G,H)** Paraffin-embedded samples from IFNAR1^−/−^ mice treated with PBS or IFN-λ_2_ as indicated were subjected to simultaneous staining for **(G)** IFIT1 (green) and E-cadherin (red) or for **(H)** MMP7 (green) and E-cadherin (red). Counterstaining was performed with DAPI (blue). White squares depict the zoomed area of the merged images in panel **(H)**. Scale 50 and 20 µm in the zoomed areas.

To confirm expression of the predominantly IFN-λ-induced genes *in vivo*, 8-week-old IFNAR1-deficient female mice were intraperitoneally stimulated with 1 µg murine IFN-λ_2_. 9 h after administration, intestinal epithelial cells were prepared and analyzed by RT-PCR. IFN-λ_2_ administration significantly enhanced expression of the prototypic ISG *Ifit1* (Figure 4A). It also significantly enhanced the expression level of the predominantly IFN-λ-induced genes *Mmp7* (Figure 4B) and *Serpinb1a* (Figure 4C). By contrast, intraperitoneal administration of 500 U IFN-β to IL-28R deficient failed to induce the prototypic ISG *Ifit1* (Figure 4D). Also, neither an increase of *Mmp7* (Figure 4E) nor *Serpinb1a* expression was observed (Figure 4F). Immunostaining subsequently confirmed induction of the prototypic ISG IFIT1 in the intestinal villus epithelium of IFN-λ_2_ treated IFNAR1-deficient animals (Figure 4G). Finally, also enhanced expression of the predominantly IFN-λ_2_-induced target MMP7 was noted in crypt based Paneth cells of IFNAR1-deficient animals following IFN-λ_2_ administration (Figure 4H).

## Discussion

The major biological difference between type I and type III IFN was shown to reside in their organ and cell-type tropism. Whereas most nucleated cells respond to type I IFN, type III IFN appears to play a non-redundant role in the protection of epithelial cells at mucosal body sites such as the gastrointestinal and respiratory tract *in vivo* (8, 9, 18). This renders type III IFNs critical components of the epithelial antiviral host response and raises the question of the evolutionary benefit of an additional epithelium-specific IFN system. First, an epithelium-specific antiviral host response acts early during the infectious challenge and may be able to cope with the microbial challenge in the absence of the well-known side effects of a systemic IFN response (39, 40). Indeed, a recent study demonstrated that the early protective IFN-λ effect occurs in the absence of significant tissue inflammation, which might be particularly important in respect to the function of the respiratory and gastrointestinal tract (22). Consistently, IFN-λ has been shown to also exert an immunomodulatory effect on PMNs (15, 16). The use of IFN-λ as an alternative therapeutic option to type I IFN has been therefore suggested for human viral hepatitis in an attempt to reduce the systemic side effects (41). Second, type III IFN may be able to simultaneously induce gene products that tailor the response to fit the needs of an anti-infectious host response at colonized mucosal surfaces.

Previous studies did not identify a type III IFN-specific gene profile (12, 20, 24–30). Notably, however, these studies employed hepatocytes or immortalized liver cell lines as well as lung epithelial cells possibly missing out on genes involved to maintain host–microbial homeostasis at the most densely colonized body surface, the intestinal tract. The striking species-specific activity of type III IFN on human but not mouse hepatocytes underlines the exceptional phenotype of hepatocytes (42). Also, our results revealed no expression of the predominantly IFN-λ-induced genes in lung epithelial cells. Intestinal epithelial cells might therefore represent the most promising cell type to investigate an IFN-λ_2_-specific cell response. Indeed, differences in the IFN receptor signal cascade have previously been observed between different cell types (12, 23, 43).

In this study, we employed a recently described immortalized intestinal epithelial cell line that exhibits a potent response to both type I and III IFN (32). These cells express a number of typical intestinal epithelial cell marker proteins and exhibit a polarized growth with increase in the transepithelial electrical resistance when cultured on porous transwell culture surfaces. Most importantly, stimulation of ISGs in IEC10 cells was induced by both, type I and III IFN in a dose-dependent manner. This cell-culture model therefore represents an ideal tool to investigate the differential response to type I versus type III IFN at the intestinal epithelial lining. In addition, we employed IL-28R and IFNAR-deficient animals in combination with protocols to isolate highly enriched primary gut epithelial cells to confirm the induction of a predominantly IFN-λ-induced gene, *Mmp7, in vivo* (9).

Comparative analysis of IFN-β versus IFN-λ_2_ stimulated IEC10 cells resulted in the identification of a predominantly IFN-λ_2_-induced gene expression profile. The identified genes do not belong to the previously defined group of classical ISGs associated with viral inhibition but their function demonstrates a clear association with the gut epithelial barrier function. MMP7 plays a critical role in tissue remodeling, encodes an immunomodulatory activity and activates Paneth cell-derived antimicrobial peptides (44). Other gene products such as the vitamin A transporter transthyretin, the ${\text{Na}}^{+}\;{\text{HCO}}_{\text{3}}^{-}$-cotransporter NBCn1 (Slc4a7), the surface membrane protein annexin A13 or the Na channel α-ENaC (encoded by Scnn1A) may contribute to metabolism, transcellular transport and ion homeostasis at the epithelium (45, 46). The dynein protein Dnah7b (dynein axonemal heavy chain 7B) the mucin-synthesis core 2 1,6-*N*-acetylglucosaminyltransferase enzyme (C2GnT-M encoded by the GCNT3 gene) and the desmosome protein premature ovarian failure 1B (Pof1b) may reinforce epithelial barrier formation (47–49). Other proteins such as the HIF1-associated regulator paired-like homeodomain pituitary transcription factor Pitx1 or the Ca dependent GTPase RAS protein activator (Rasal1) may be involved to tailor cellular functions and epithelial gene expression (50, 51). Thus, enhanced expression of predominantly IFN-λ_2_-induced gene products may help to control the inflammatory reaction at impaired mucosal body sites and reconstitute the epithelial barrier integrity and host–microbial homeostasis following viral clearance.

This hypothesis is also consistent with the fact that type III IFNs belong to the IL-10 cytokine family, a large group of cytokines that also includes IL-10, IL-19, IL-20, IL-22, IL-24, and IL-26. Members of this family play a critical role in the maintenance and repair of the epithelial barrier function during infectious and inflammatory challenges (52). They exhibit a strong immunomodulatory activity illustrating the adverse effect of uncontrolled mucosal inflammation and the need to maintain the integrity of body surfaces and host–microbial homeostasis. This is nicely illustrated by IL-10 that is able to repress pro-inflammatory responses playing a critical role to maintain mucosal homeostasis in the colon (53). Also, IL-22 strengthens the mucosal barrier and induces antibacterial effector molecules in the absence of an inflammatory response. Of note, IL-22 and type III IFN were recently shown to synergize to restrict viral replication at the intestinal epithelium (54).

Cell polarization appears to play a critical role for the expression of the predominantly IFN-λ_2_-induced gene profile. Apical–basolateral polarization represents a key feature of intestinal epithelial cells and has previously been functionally associated with the response of gut epithelial cells to IFN (9). Strikingly, epithelial cell polarization significantly enhanced the expression level of the IL-28Rα chain but not of the IFNAR receptor complex confirming a previous report (37). It is therefore tempting to speculate on a possible functional link between the level of expression of the type III IFN receptor and the ability to induce additional cellular signal transduction pathways ultimately inducing a predominantly IFN-λ_2_-induced gene profile. Alternatively, the cell polarization itself may influence downstream events of the IL-28 receptor complex. Future investigations will be needed to identify and dissect the involved signaling pathways.

In conclusion, we here report on the first evidence for the existence of a predominantly IFN-λ_2_-induced gene expression profile in polarized intestinal epithelial cells *in vitro* and *in vivo*. Expression of predominantly IFN-λ_2_-induced genes was restricted to gut epithelial cells and required apical–basolateral cell polarization. The existence of a predominantly IFN-λ-induced gene set at the intestinal epithelium might significant extend the biological role of IFN-λ and shed light on the particular situation at microbially colonized mucosal surfaces during infectious challenges.

## Ethics Statement

All animal experiments were performed in compliance with the German animal protection law (TierSchG) and approved by the local animal welfare committee Niedersächsisches Landesamt für Verbraucherschutz und Lebensmittelsicherheit Oldenburg, Germany. Mice were housed under specific pathogen-free conditions and handled in accordance with regulations defined by FELASA and the national animal welfare body GV-SOLAS (www.gv-solas.de/index.html).

## Author Contributions

TS, SB, MK, and MH performed experiments. TS, SB, MK, DW, HH, and MH planned the experiments and evaluated the results. HH, MK, and UK provided critical reagents. TS, MK, DW, HH, UK, and MH wrote the manuscript.

## Conflict of Interest Statement

The authors declare that the research was conducted in the absence of any commercial or financial relationships that could be construed as a potential conflict of interest.

## Footnotes

^1^www.gv-solas.de.

^2^http://www.pantherdb.org/.

## Supplementary Material

Figure S1 Interferon (IFN) effect on house-keeping gene expression and time kinetic of IFN-β and IFN-λ_2_-induced IFN-stimulated gene induction. **(A)** mRNA expression (intensity) of the house-keeping gene *β-actin* in unstimulated IEC10 cells (control) and IEC10 cells stimulated with IFN-β (500 U/mL) and IFN-λ_2_ (20 ng/mL) for 9 h. The results represent the mean ± SEM values from one experiment performed in triplicates. **(B)** IEC10 cells carrying an Mx2-luciferase reporter were stimulated with IFN-β (500 U/mL) and IFN-λ_2_ (20 ng/mL) for the indicated time periods and the luciferase production was determined. The data are presented as mean ± SD from one experiment performed in triplicates.

Figure S2 Gene induction by interferon (IFN)-λ_2_ in IEC10 cells. **(A)** Heatmap of stimulated genes in IEC10 cells cultured on transwell filter inserts and left untreated (PBS) or exposed to IFN-λ_2_ (20 ng/mL) or IFN-β (500 U/mL) for 9 h. Data were obtained using a global gene expression array. Two-group analysis for the induction of IFN-λ_2_-induced genes were carried out at *p* = 0.001, *q* = 0.05, FC = 2 represented as a hierarchical cluster of 349 genes upregulated by IFN-λ_2_. **(B–D)** Clusters of orthologous group analysis of the **(B)** “predominantly IFN-λ_2_-induced genes,” **(C)** “strongly IFN-λ_2_-induced genes,” and **(D)** “classical antiviral IFN-stimulated genes,” shown in Figure 1C.

Figure S3 Early time kinetic of interferon (IFN)-β and IFN-λ_2_-induced IFN-stimulated gene (ISG) induction. **(A,B)** Quantitative RT-PCR for the prototypical ISGs *Usp18* and *Ifi44* as well as the predominantly IFN-λ_2_-induced ISGs *Mmp7* and *Serpinb1a* performed on total RNA isolated from IEC10 cells grown on transwell filter inserts and stimulated with **(A)** IFN-β (500 U/mL) and **(B)** IFN-λ_2_ (20 ng/mL) for the indicated time periods. The results represent the mean ± SEM values from one experiment performed in triplicates and are normalized to the values obtained for the housekeeping gene *β-actin*.
